# Oral white sponge nevus in patients with ectrodactyly-ectodermal dysplasia-cleft syndrome: two patients treated with liquid nitrogen cryotherapy^؆^

**DOI:** 10.1016/j.abd.2026.501401

**Published:** 2026-06-17

**Authors:** Paula Gerlero, Silvia Vanessa Lourenço, Marcello Menta Simonsen Nico

**Affiliations:** a Department of Dermatology, Faculty of Medicine, Universidade de São Paulo, São Paulo, SP, Brazil; b Department of Pathology, Faculty of Dentistry, Universidade de São Paulo, São Paulo, SP, Brazil

Dear Editor,

Ectrodactyly-Ectodermal Dysplasia-Cleft (EEC) syndrome is an extremely rare genetic disorder. Although the exact prevalence is unknown, 300 cases have been reported.^1^ In over 90% of these cases, EEC syndrome is due to missense mutations in the *TP63* gene (3q27), essential for ectoderm and limb development.^1^ EEC syndrome presents a wide intra- and interfamilial clinical variability, and cardinal signs may occur with varying degrees of severity.^1^, ^2^ The syndrome is defined by three cardinal signs: ectrodactyly, (oligodactyly and syndactyly of the hands and feet), ectodermal dysplasia, and cleft lip (with or without cleft palate). Both ectodermal and mesodermal tissues may be affected, resulting in a spectrum of phenotypes.

White Sponge Nevus (WSN) is a rare autosomal dominant keratinopathy^3^ with variable expressivity and irregular penetrance, affecting approximately one in 200,000 individuals.^4^ In a retrospective study, four cases of WSN were found in the last 30-years, representing 0.02% from a total of 14108 cases evaluated in this period.^5^ WSN results from defects in the keratinization process of oral mucosal epithelium and has been associated with heterozygous missense mutations in CK13 and CK4.^6^, ^7^ The oral mucosa is affected in the majority of cases. Diagnosis of WSN is usually clinical: white, gray, diffuse, thickened, corrugated or velvety plaques, which do not disappear upon stretching the tissue. A biopsy may be required to exclude other conditions. Microscopic features of oral WSN include hyperparakeratosis, acanthosis, papillomatosis and perinuclear eosinophilic condensations of the keratinocytes associated with clearing of the cytoplasm.

We report two unrelated patients with ECC syndrome and WNS, and the results of treating symptomatic lesions of WNS with cryotherapy. This retrospective case series was conducted at the Department of Dermatology, Hospital das Clínicas, University of São Paulo, Brazil. The research was IRB approved.

Patient 1 was an 11-year-old Brazilian boy diagnosed with EEC syndrome at birth, with a positive family history, as his mother was also affected. Clinical findings included ectrodactyly with syndactyly (Fig. 1A), keratoconjunctivitis, sparse hair, and hypodontia and microdontia. Oral examination revealed diffuse and generalized white colored oral mucosal thickening (Fig. 1B). Lesions extended to the labial commissures, becoming dry and fissured, with pain (Fig. 1C). Histopathological analysis was consistent with WSN. The patient requested treatment for the discomfort of labial commissure lesions. Methodology consisted of intradermal anesthesia with lidocaine, followed by 10-second open spray liquid nitrogen applications with a CryAc® cryotherapy device. After three monthly sessions, WSN was totally cleared, with complete resolution of fissuring and pain without relapse after 12-years (Fig. 1D).

**Fig. 1:**
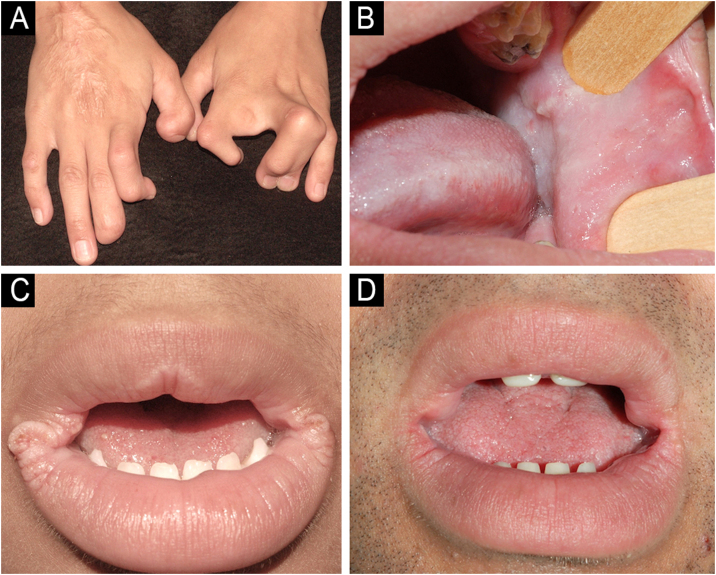
(A) Ectrodactily and sindactily; (B) WSN: white thickening of the oral mucosa and lateral tongue; (C) WSN on the labial commissures ‒ papillomatous aspect with fissures; (D) Twelve years after cryotherapy, commissural lesions did not relapse.

Patient 2 was a 23-year-old Brazilian man with EEC syndrome presenting fissured lip (surgical correction before), lacrimal duct obstruction, oligodactyly of the hands (Fig. 2A), sparse hair, and oral changes consistent with WSN. Lesions diffusely affected the oral mucosa, including tongue, labial commissures and lower lip vermilion where a keratotic, papillomatous, and fissured appearance developed, associated with pain and bleeding (Fig. 2B). Cryotherapy was performed as on Patient 1. Results were equally positive, and lesions did not relapse after ten years (Fig. 2C). Histopathological analysis was consistent with WSN (Fig. 3A‒B).

**Fig. 2:**
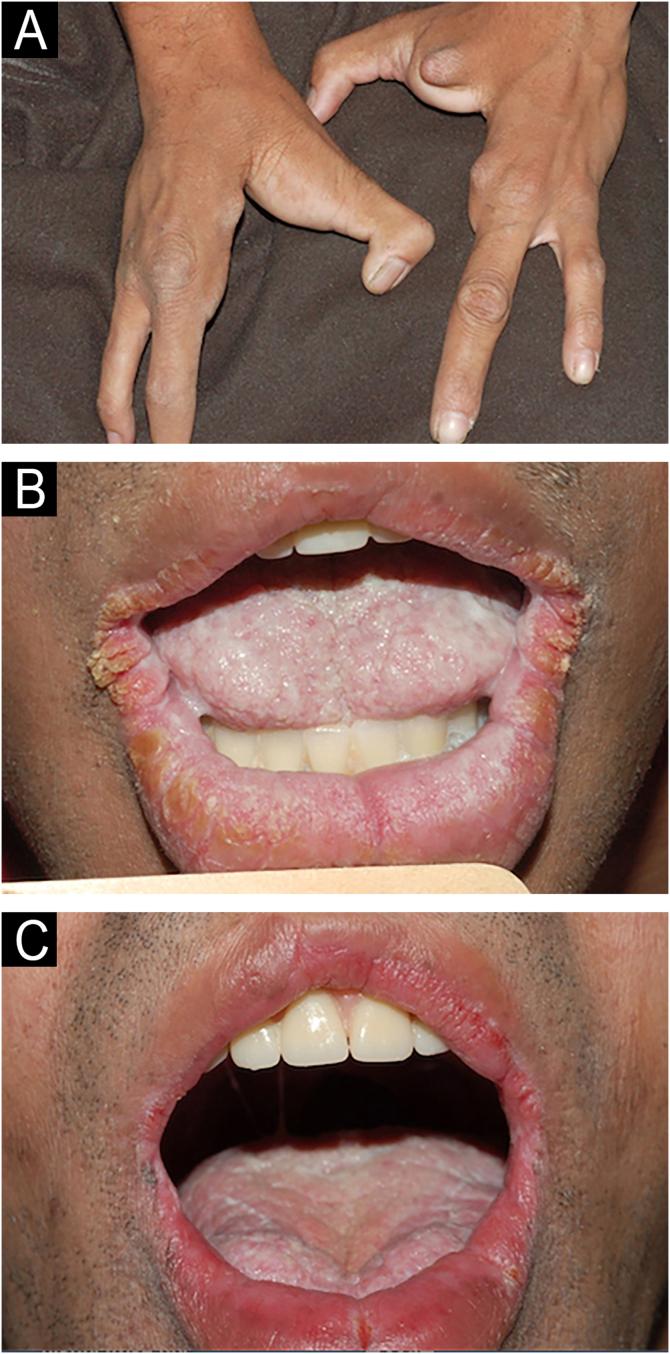
(A) Oligodactyly; (B) WSN: thickening of the lingual mucosa; keratosis and fissures on the commissures and on the lower lip vermilion; (C) Excellent result with cryotherapy; this picture was taken several years after treatment.

**Fig. 3:**
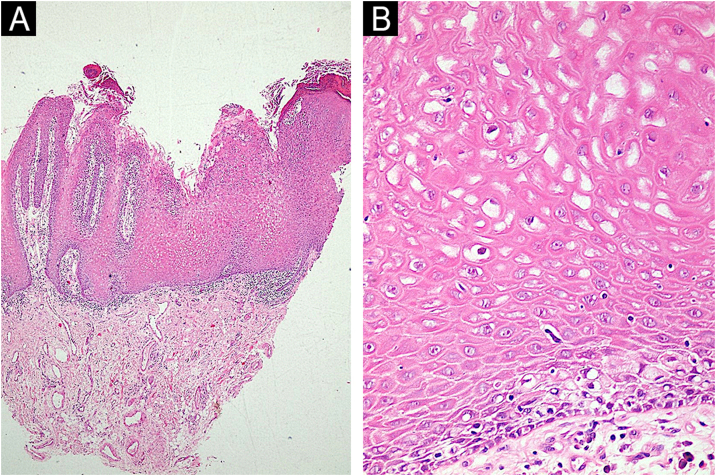
Histopathology of WSN. (A) Acanthosis, papillomatosis, clear spaces in the mucosal epithelium; (B) Cytoplasmatic clearing and eosinophilic perinuclear condensations of the mucosal keratinocytes (Hematoxylin & eosin, a‒100×, b‒400×).

The association between EEC syndrome and WSN is extremely rare and poorly studied. To our knowledge, one case of WSN has been reported in a patient with EEC syndrome, and we believe that the changes were subtle and can be attributed to cheek biting.^8^ In contrast, both patients in the present report exhibited significant diffuse mucosal thickening, including the tongue and palate. The presence of characteristic perinuclear condensations of the mucosal keratinocytes suggests a shared keratin-related pathogenic mechanism, similar to that observed in other keratin disorders.^3^, ^9^ We also believe that the association of WSN with EEC in patients, although seldom described, might not be fortuitous, since manifestations were almost identical in both patients. Cryotherapy was highly effective in the external hyperkeratotic lesions with excellent cosmetic and functional results. We opted not to treat the intraoral lesions of WSN in our patients since they were completely asymptomatic.

Limitations of our study included its retrospective design and results from a single academic center. The absence of genetic sequencing also represents a limitation, as molecular confirmation was not available.

In conclusion, this case report is presented for its rarity, reporting two additional cases of WSN with emphasis on clinical and histopathological features, besides the rare association with EEC syndrome and the excellent response with cryotherapy treatment.

Considering the low prevalence of both disorders, future studies on their etiology might look at the common etiopathogenic aspects.

## Authors’ contributions

Paula Gerlero: Data analysis and interpretation; active participation in research supervision; intellectual participation in the therapeutic management of the case studies; final approval of the final version of the manuscript.

Silvia Vanessa Lourenço: Data analysis and interpretation; active participation in research supervision; intellectual participation in the therapeutic management of the case studies; final approval of the final version of the manuscript.

Marcello Menta Simonsen Nico: Study conception and design; data analysis and interpretation; critical review of important intellectual content; active participation in research supervision; intellectual participation in the therapeutic management of the case studies; final approval of the final version of the manuscript.

## Financial support

None declared.

## Research data availability

Does not apply.

## Conflicts of interest

None declared.
